# Proportion of toxin and non-toxin virulence factors of *Staphylococcus aureus* isolates from diabetic foot infection: a systematic review and meta-analysis

**DOI:** 10.1186/s12866-023-03142-y

**Published:** 2024-01-03

**Authors:** Samaneh Shahrokh, Aliye Tabatabaee, Maryam Yazdi, Mansour Siavash

**Affiliations:** 1 Isfahan Endocrine and Metabolism Research Center, Isfahan University of Medical Sciences, Isfahan, Iran; 2 Child Growth, and Development Research Center, Research Institute for Primordial Prevention of Non-Communicable Diseases, Isfahan University of Medical Sciences, Isfahan, Iran

**Keywords:** Staphylococcus aureus, Virulence factors, Diabetic foot, Foot ulcer, Infections

## Abstract

**Background:**

*Staphylococcus aureus* isolates are the leading cause of diabetic foot infections (DFIs). Identification of specific virulence factors of *S. aureus* involved in the pathogenesis of DFIs may help control the infection more effectively. Since the most prevalent virulence factor genes are probably related to the DFI pathogenesis, the aim of this study is to evaluate the proportion of virulence factor genes of *S. aureus* isolates from DFIs.

**Materials and methods:**

We conducted a systematic search of PubMed, Embase, Web of Science, and Scopus to identify all articles reporting the proportion of different types of virulence factors of *S. aureus* isolates from DFI samples.

**Results:**

Seventeen studies were eligible, in which 1062 *S. aureus* isolates were obtained from 1948 patients and 2131 DFI samples. Among the toxin virulence factors, *hld* 100.0% (95% CI: 97.0, 100.0%), *hlg* 88.0% (95% CI: 58.0, 100.0%), *hla* 80.0% (95% CI: 31.0, 100.0%), *hlgv* 79.0% (95% CI: 35.0, 100.0%) and *luk-ED* 72.0% (95% CI: 42.0, 95.0%) had the highest proportion respectively. Among the genes associated with biofilm formation, both *icaA* and *icaD* had the highest proportion 100.0% (95% CI: 95.6, 100.0%).

**Conclusion:**

The results of the present study showed that among the toxin virulence factors, hemolysins (*hld*, *hlg*, *hla*, *hlgv*) and *luk-ED* and among the non-toxin virulence factors, *icaA* and *icaD* have the greatest proportion in *S. aureus* isolates from DFIs. These prevalent genes may have the potential to evaluate as virulence factors involved in DFI pathogenesis. Finding these probable virulence factor genes can help control diabetic foot infection more effectively via anti-virulence therapy or preparation of multi-epitope vaccines.

**Supplementary Information:**

The online version contains supplementary material available at 10.1186/s12866-023-03142-y.

## Introduction

*Staphylococcus aureus* is a leading cause of serious infections with high morbidity, mortality and health-related costs. *Staphylococcus aureus* can cause a variety of clinical diseases via various potential virulence factors. These diseases include bacteremia, endocarditis, osteomyelitis, as well as skin and soft tissue, osteoarticular, pulmonary and device-related infections [1]. In a systematic review and meta-analysis, it was reported that the mortality rate from *S. aureus* bacteremia was 18.1%, 27.0%, and 30.2% at 1 month, 3 months, and 1 year, respectively [2]. *S. aureus* is also the leading invasive bacterial pathogen in children in many parts of the world [3].

In particular, *S. aureus* is one of the most common bacteria isolated from diabetic foot infections (DFIs) worldwide. In our recent systematic review and meta-analysis, we reported that the highest pooled proportion of isolated bacteria from DFIs in Iran belongs to *S. aureus* (24.29%), of which 55% were methicillin resistant strains (MRSA) [4].

Fighting this leading bacterium presents two major challenges. The first problem is that *S. aureus* expresses many potential toxin and non-toxin virulence factors that intensively target many surfaces and tissues. The second problem is the increasing resistance of *S. aureus* isolates from DFIs to the most commonly prescribed antibiotics. In fact, MRSA has emerged as one of the major epidemiological and clinical problems [5].

Toxin virulence factors are classified into pore-forming toxins, exfoliative toxins, enterotoxins and epidermal cell differentiation inhibitor toxins. The pore-forming toxins include the single-component α-toxin (α-hemolysin), the phenol-soluble modulins (PSMs), and bi-component leukotoxins, including Panton-Valentine leukocidin (PVL), γ-hemolysin, and leukocidin E/D [6]. Some of the non-toxin virulence factor genes are involved in biofilm formation, such as: *icaA*, *icaD* and *atl* as well as *pls*. *S.aureus* produces surface proteins called MSCRAMM (Microbial Surface Components Recognizing Adhesive Matrix Molecules) and mediates adhesion to the ulcer surface [7]. Typical members of the MSCRAMM family are staphylococcal protein A (SpA), collagen-binding protein, fibronectin-binding proteins A and B (FnbpA and FnbpB), and clumping factor proteins (Clf) A and B [8].

It is the time to focus on new antimicrobial agents for resolving the above-mentioned problems. Among the new therapeutic strategies, anti-virulence therapy has emerged as a new promising strategy [9]. In this method, instead of fighting the bacteria, their pathogenic virulence factors are targeted [9]. Unlike conventional antibiotics, this method may cause lower selective pressure over pathogens and therefore lower emergence and spread of resistance [9].

Given the wide range of different virulence factors mentioned above, an important question arises as to which of these factors of *S. aureus* can be specifically related to DFI pathogenesis. Several studies measured and characterized the virulence factors of *S. aureus* isolates from DFIs [10–26]. and some of them introduced potential virulence factors to distinguish colonization from infection [10–12].

Since the identification of the most prevalent virulence factor genes of infecting *S. aureus* isolates may be related to both their pathogenesis and the differentiation between colonization and infection, the aim of this systematic review and meta-analysis is to evaluate the proportion of virulence factor genes of *S. aureus* isolates from DFIs.

## Materials and methods

### Study protocol

This systematic review and meta-analysis was conducted in accordance with the PRISMA (Preferred Reporting Items for Systematic Reviews and Meta-Analyses) guidelines [27] and a PRISMA checklist was completed. The study protocol has been registered at the Isfahan University of Medical Sciences with the national ethics code of IR.MUI.MED.REC.1399.450.

### Data sources and searching strategy

We ran a thorough search in PubMed, Embase, Web of Science, and Scopus following Mesh terms and keywords: ‘virulence’, ‘pathogenicity’, “pathogenicity factor*”, “virulence factor*”, “virulence gene*” And “Staphylococcus aureus”, “S. aureus” And “diabetic foot”, “diabetic feet” And ‘ulcer’, ‘infection’, ‘wound’, ‘osteomyelitis’, ‘cellulitis’, ‘abscess’, ‘gangrene’. There was no publication date and language limit/restriction.

### Inclusion and exclusion criteria

This systematic review included original laboratory-based cross-sectional prevalence studies that measured at least one virulence factor gene of *S. aureus* isolates from human-infected diabetic foot ulcers (grade 2–4). We also excluded all reviews and studies that used animal infections.

### Screening and eligibility of studies

The study procedure was carried out by two independent reviewers. Any disagreements were discussed between these reviewers or consulted with a third reviewer. After removing duplicate publications, titles and abstracts of the remaining articles were reviewed for potentially eligible studies. The full text of the remaining studies was then assessed for eligibility. Studies that met the inclusion criteria were considered eligible and were included in the present study. One reviewer extracted the data and a second reviewer verified its accuracy. The following data were extracted: author name, publication date, country, ulcer classification, molecular methods, number of patients, number of DFUs, number of *S. aureus* isolates, and frequency of each virulence factor.

### Critical appraisal of studies

The quality of selected studies was evaluated using standard critical appraisal tools prepared by the Joanna Briggs Institute (JBI) for prevalence studies [28]. The purpose of this appraisal is to assess the methodological quality of a study and to determine the extent to which a study has addressed the possibility of bias in its design, conduct, and analysis. The JBI critical appraisal checklist contains nine questions (Q1-Q9). The scores given by two reviewers were used to make the final decision. A third reviewer was consulted in case of disagreement between their appraisal opinions. Studies with five or more “YES” responses (55% YES response rate) were included in the meta-analysis.

### Virulence factor measurements

In the first step, we constructed a list of *S. aureus* virulence factor genes by precise examining all included studies and studying several reviews and related original articles [6, 8, 29, 30]. For better analysis, we divided the virulence factor genes of *S. aureus* into two categories: toxin and non-toxin. Toxin and non-toxin virulence factors mentioned in at least three or more studies were included in the meta-analysis. The outcome of interest was the number of isolates possessing each virulence factor gene.

### Statistical analysis

The point estimates of the proportion of each virulence factor and its 95% confidence interval (95% CI) were estimated for each study. To estimate the pooled proportions, we used Metaprop, a statistical procedure in STATA (version 14) [31]. A random-effects model including Freeman-Tukey double arcsine transformation of the proportions was used to stabilize variance and reduce the effect of between-study heterogeneity. 95% CIs were computed around study-specific and pooled prevalence of each virulence factor based on the score test statistic and visualized by forest graphs. Between- study heterogeneity was evaluated with Cochran’s Q-test [32] and the percentage of total variation across studies was assessed with the I² measure [33]. Publication bias was tested by Begg’s test, and funnel plot. P values less than 0.05 were considered as statistically significant.

## Results

### Study selection

**Fig. 1:**
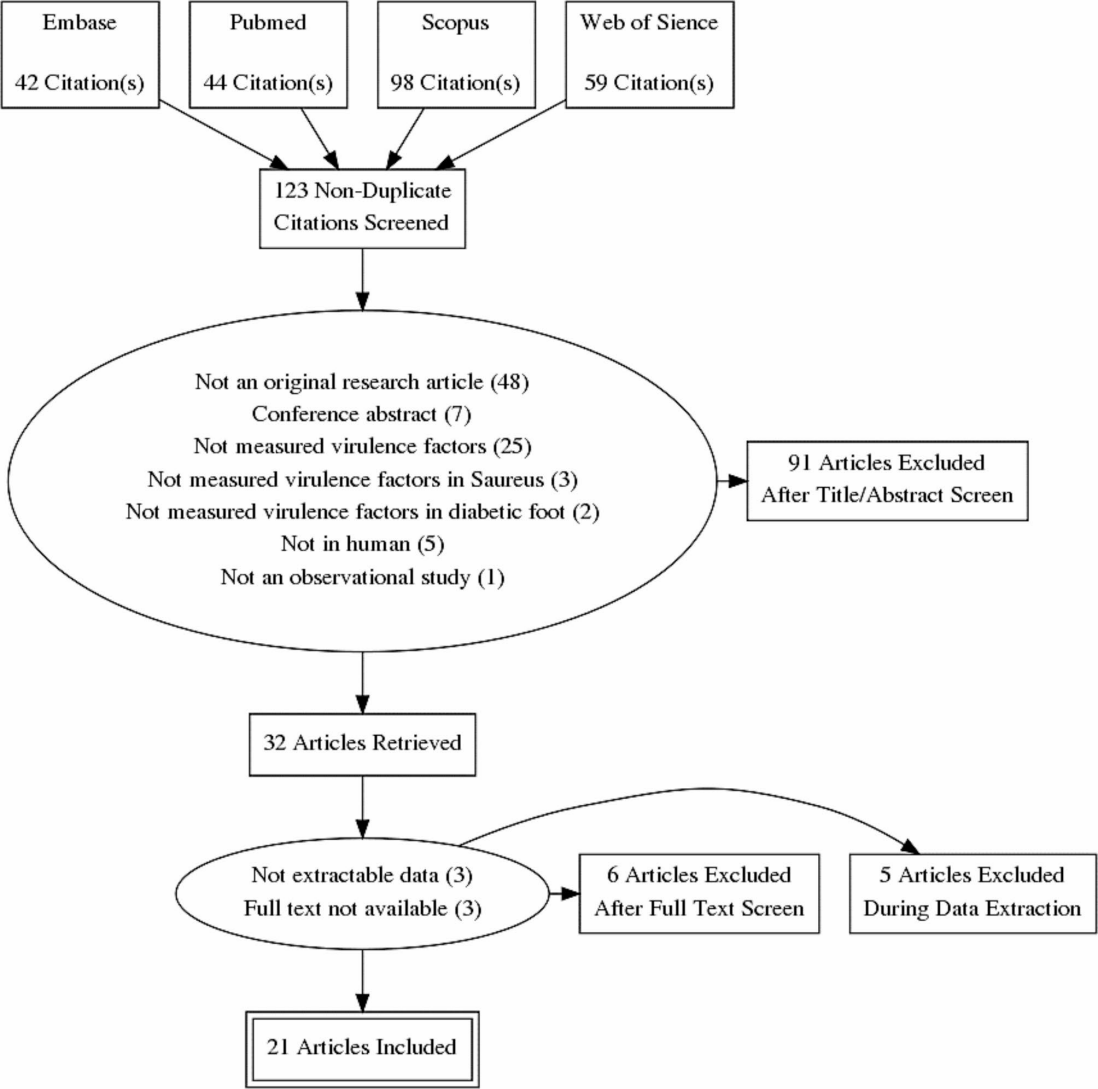
Preferred reporting items for systematic reviews and meta-analyses (PRISMA) flow diagram depicting the selection process

A literature search in electronic databases including PubMed, Embase, Web of Science and Scopus retrieved a total of 243 articles. After removing duplicates (n = 120), 91 studies were excluded in the initial screening of titles and abstracts. Subsequently, 13 additional articles were removed in full-text screening. Twenty-one articles met all eligibility criteria and were included in the systematic review study. Inter-rater agreement between reviewers for study selection was excellent (Kappa statistics = 0.96). The study selection process is detailed in Fig. 1.

### Characteristics of included studies

A total of 1062 *S. aureus* isolates from 1948 patients were examined using 2131 DFI samples. The number of *S. aureus* isolates ranged from 8 [14] to 195 [12]. The number of virulence factor types measured in one study ranged from one [23, 24] to more than thirty [11, 13, 14, 20]. It is interesting that four continents, including Europe (9), Asia (6), Africa (1) and North America (1) had contribution in this topic. Among countries, France contributed the most with five (23.8%) publications [12, 19–22]. All included studies were published within the last 15 years. Six studies [11, 14–18, 24, 25] did not report a clear ulcer classification system. Most articles used PCR methods to measure virulence factor genes (Table 1).

**Table 1:** Characteristics of the included studies in this systematic review and meta-analysis

First author (reference)	Year	Country		Design	No. of patients	No. of DFI samples	No. of isolates	Ulcer classification system	Methods used for determination of virulence factors	No. of measured virulence factor types
Sotto, et al. [10]	2007		France	Prospective study	72	72	85	IDSA	Oligonucleotide DNA arrays & PCR	3
Sotto, et al. [11]	2008		France	Prospective longitudinal study	118	118	132	NR	PCR	33
Sotto, et al. [12]	2012		France	Prospective study	195	195	195	IDSA/IWGDF	Oligonucleotide DNA arrays	23
Djahmi, et al. [13]	2013		Algeria	Prospective study	128	183	85	IDSA-IWGDF	Oligonucleotide DNA arrays	33
Post, et al. [15]	2014		Switzerland & France	Retrospective study	23	23	23	NR	PCR	21
Paul, et al. [14]	2014		Bangladesh	NR	8	8	8	NR	Multiplex PCR	36
Stappers, et al. [16]	2015		Netherlands	RCT	NR	128	113	NR	Real-time PCR	2
Shettigar, et al. [19]	2015		India	Prospective study	200	200	86	IDSA-IWGDF	Multiplex PCR	3
Mottola, et al. [17]	2016		Portugal	Transversal observational study	49	49	41	NR	PCR	9
Pobiega, et al. [18]	2016		Poland	Laboratory-based study	68	68	68	NR	PCR	9
Dunyach-Remy, et al. [20]	2017		France	Prospective study	276	276	65	IDSA–IWGDF	Oligonucleotide DNA arrays	37
Víquez-Molina, et al. [21]	2018		Costa Rica	Cross-sectional exploratory study	379	379	58	IDSA	PCR	4
Kananizadeh, et al. [22]	2019		Iran	Cross-sectional study	145	145	30	Wagner	Multiplex PCR	2
Silva, et al. [25]	2020		Portugal	NR	42	42	25	Wagner	PCR	8
Anwar, et al. [23]	2020		Iraq	cross-sectional	46	46	24	IWGDF	Multiplex PCR	1
Lin, et al. [24]	2020		Taiwan	NR	112	112	10	IDSA	PFGE	1
Al-Bakri, et al. [26]	2021		Jordan	cross-sectional	87	87	14	Wagner	Multiplex PCR	8

### Results of quality assessment

Quality of the studies was assessed using JBI tool. Seventeen out of 21 articles received at least five “YES” answers and were included in the meta-analysis (Table S1).

### Virulence factor measurements

Among 17 included articles, 15 and 9 articles measure toxin and non-toxin virulence factors respectively. Seven articles reported both toxin and non-toxin virulence factors [11–14, 16, 20, 25]. The following virulence factors were measured in three or more studies, and they were included in the meta-analysis: 24 toxin virulence factors (*hla, hlb, hlg, hlgv, hld, luk-SF* or *PVL, luk-ED, etA, etB, etD, sea, seb, sec, sed, see, seg, seh, sei, sej, sek, seq, tst, edin-A, edin-B*) and 19 non-toxin virulence factors (*bbp, cna, ebpS, clfA, clfB, fib, fnbA, fnbB, eno, cap5, cap8, agr 1, agr 2, agr 3, agr 4, icaA, icaD, chp, scn*).

### Toxin virulence factors

**Fig. 2:**
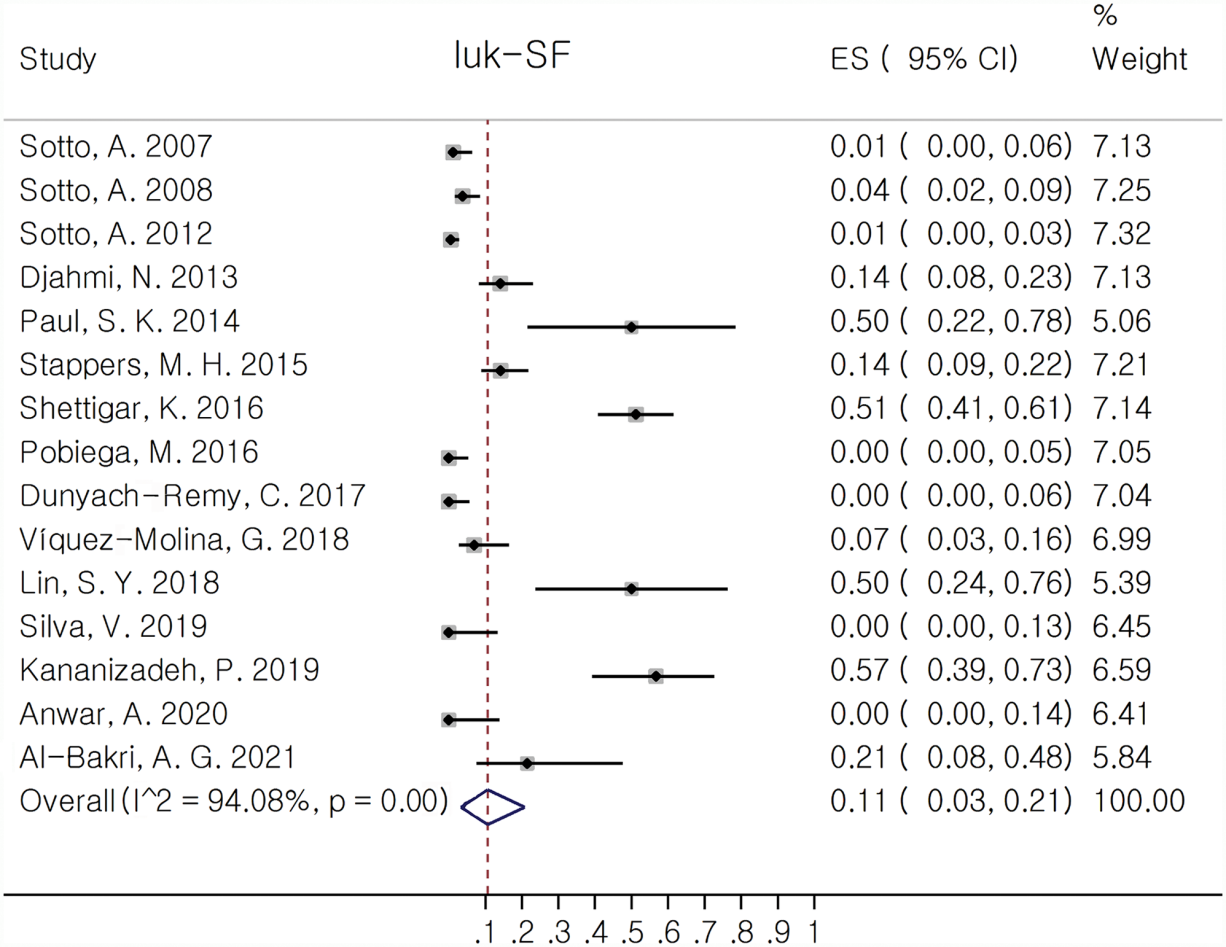
Forest plot showing the pooled proportion of luk-SF (PVL) in *Staphylococcus aureus* isolates from diabetic foot infection (DFI)

**Fig. 3:**
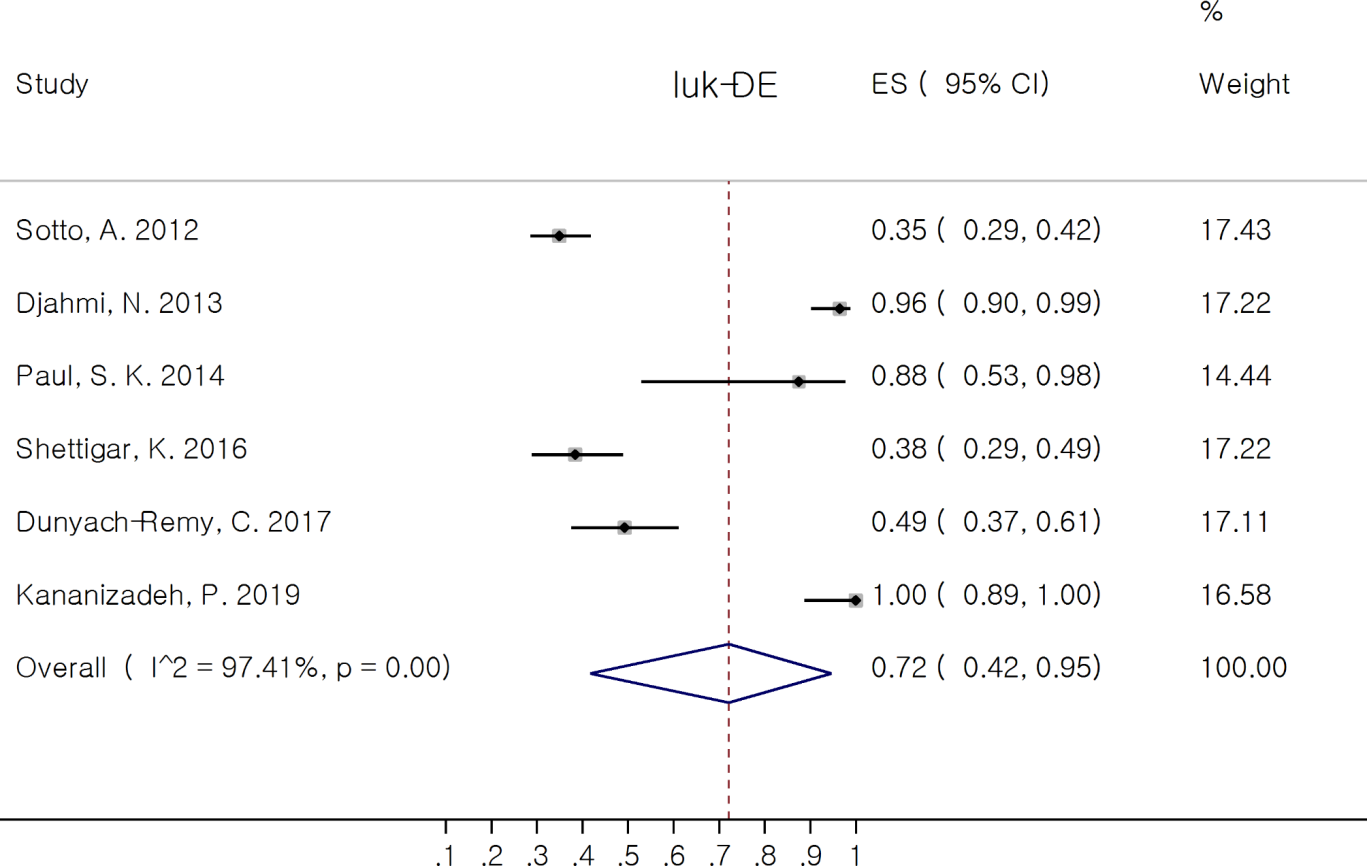
Forest plot showing the pooled proportion of luk-ED in *Staphylococcus aureus* isolates from diabetic foot infection (DFI)

luk-SF (PVL) was the most prevalent reported virulence factor since it was reported in 15 out of 17 included studies. Among pore forming toxins, Bi-Component Leukotoxins had the most contribution. In this group, *hld* 100.0% (95% CI: 97.0, 100.0%) and *hlg* 88.0% (95% CI: 58.0, 100.0%) had the most and *luk-SF* (PVL) 11.0% (95% CI: 3.0, 21.0%) the least pooled estimate of proportion. Among leukocidin family, *luk-ED* had the most pooled proportion 72.0% (95% CI: 42.0, 95.0%). The corresponded forest plots of *luk-SF* and *luk-ED* are depicted in Figs. 2 and 3 respectively.

The proportion of toxin virulence factors of *S. aureus* isolates are reported in Table 2. The prevalence of Exfoliative Toxins (etA, etB and etD), tst, and Epidermal Cell Differentiation Inhibitors Toxins (edinA, edinB) was near zero. The proportion of all Staphylococcal Enterotoxins were below 30%. Among them seg 28.9% (95% CI: 12.9, 47.9%) and sea 28.2% (95% CI: 17.9, 39.7%) had the most and seh 2.5% (95% CI: 0.0, 7.4%) and see 0.0% (95% CI: 0.0, 0.0%) the least pooled estimate of proportion.

**Table 2:** Meta-analysis for the proportion of toxin virulence factors of *S. aureus* isolates from DFIs

Toxin virulence factor genes				Pooled estimates			Heterogeneity test	
	N	n_**1**_	n_**2**_	Proportion (%)	95% CI (%)		I^2^ (%)	
*hla*	4	183	118	80.0	(31.0,100.0)		97.5	
*hlb*	4	360	135	47.0	(29.0,66.0)		87.8	
*hlg*	5	304	237	88.0	(58.0,100.0)		96.6	
*hlgv*	5	485	278	79.0	(35.0,100.0)		98.9	
*hld*	3	158	157	100.0	(97.0,100.0)		51.6	
*luk-SF or PVL*	15	998	86	11.0	(3.0,21.0)		94.1	
*luk-ED*	6	469	222	72.0	(42.0,95.0)		97.4	
*etA*	7	611	7	0.2	(0.0,1.3)		21.3	
*etB*	6	543	0	0.0	(0.0,0.0)		0.0	
*etD*	4	353	19	3.8	(0.0,11.6)		79.1	
*sea*	8	652	173	28.2	(17.9,39.7)		87.5	
*seb*	6	372	23	4.5	(0.4,11.2)		75.5	
*sec*	3	208	24	11.3	(2.0,25.1)		75.7	
*sed*	3	205	52	13.7	(0.2,38.5)		90.7	
*see*	3	154	0	0.0	(0.0,0.0)		0.0	
*seg*	4	219	79	28.9	(12.9,47.9)		80.5	
*seh*	6	372	11	2.5	(0.0,7.4)		66.6	
*sei*	4	403	120	27.4	(16.5,39.8)		79.9	
*sej*	3	208	35	8.8	(0.0,40.8)		95.1	
*sek*	5	375	75	19.2	(0.0,55.3)		98.0	
*seq*	4	290	72	19.3	(0.0,67.8)		98.4	
*tst*	8	558	60	9.6	(4.1,16.9)		81.6	
*edin-A*	3	166	0	9.6	(4.1,16.9)		81.6	
*edin-B*	3	158	12	4.9	(0.0,23.0)		87.1	

N: number of studies, n1: total number of isolates in all of the studies that report the respective virulence factor; n2: sum of the number of the isolated bacteria that report the respective virulence factor; CI: confidence interval

### Non-toxin virulence factors

**Fig. 4:**
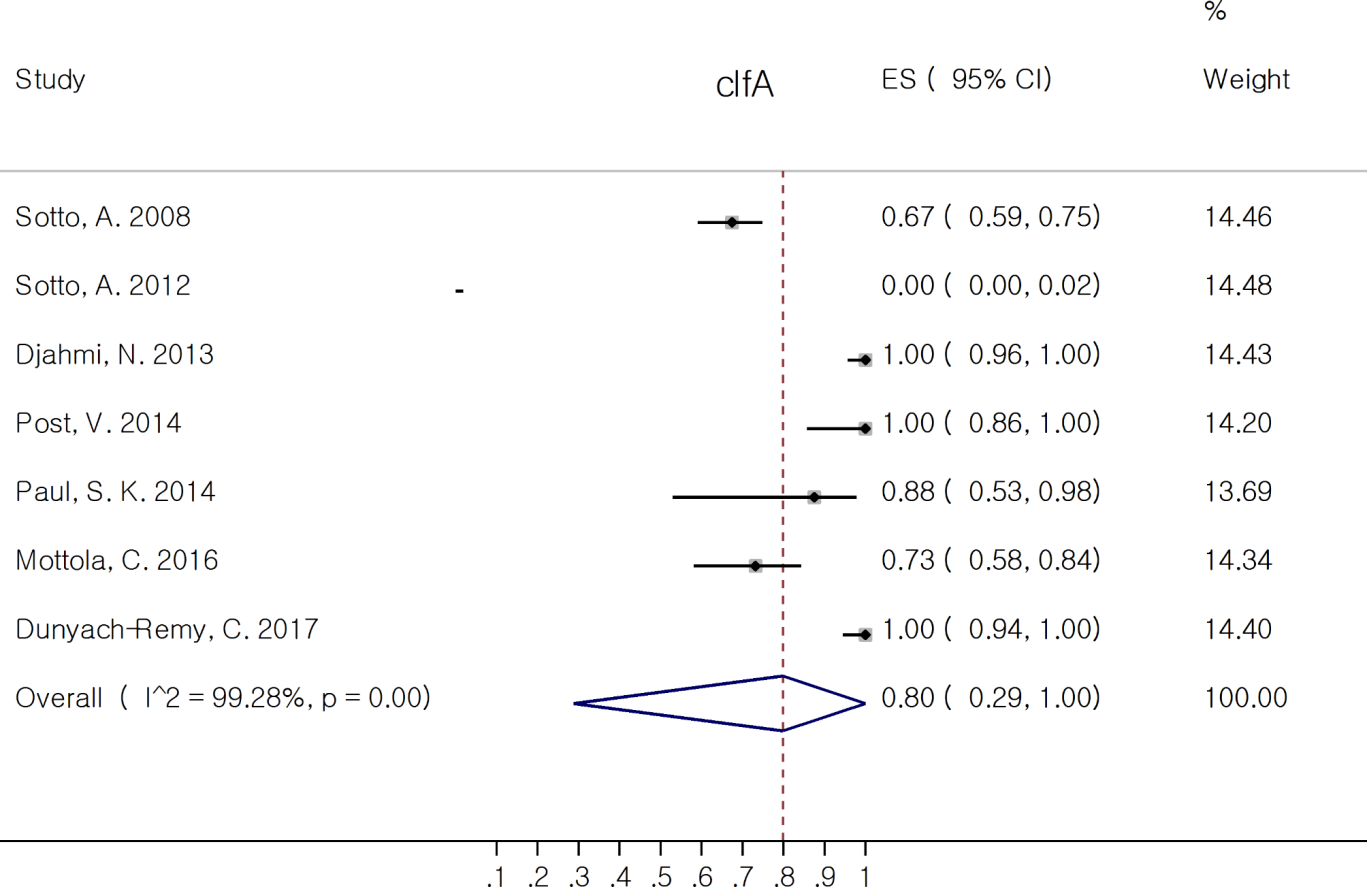
Forest plot showing the pooled proportion of clfA in *Staphylococcus aureus* isolates from diabetic foot infection (DFI)

**Fig. 5:**
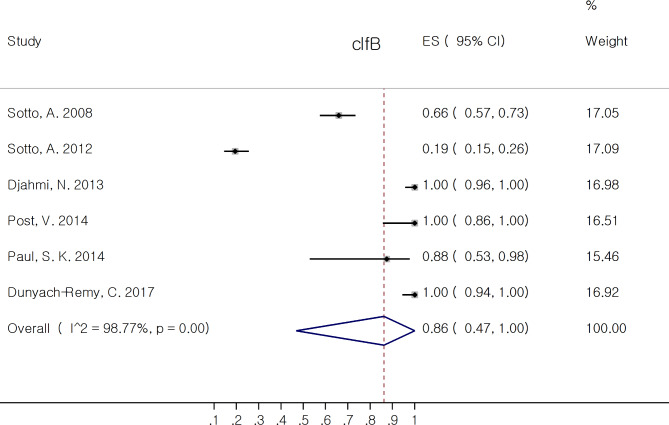
Forest plot showing the pooled proportion of clfB in *Staphylococcus aureus* isolates from diabetic foot infection (DFI)

The proportion of non-toxin virulence factors of *S. aureus* isolates are reported in Table 3. Among MSCRAMM (*bbp, cna, ebpS, clfA, clfB, fib, fnbA, fnbB*), *clfa* 79.8% (95% CI: 28.8, 100.0%) and *clfb* 86.2% (95% CI: 46.9, 100.0%) had the most pooled prevalence. The correspond forest plots of *clfA* and *clfB* are depicted in Figs. 4 and 5, respectively. Six out of eight virulence factors (*bbp, ebpS, clfA, clfB, fib*, and *fnbA*) had pooled rate of proportion above 50%. Among genes associated with biofilm formation, both *icaA* and *icaD* 100.0% (95% CI: 95.6, 100.0%) had the most pooled estimate of proportion. Among *agr* type, agr1 38.2% (95% CI: 17.7, 60.9%) had the most pooled prevalence.

**Table 3:** Meta-analysis for the proportion of non-toxin virulence factors of *S. aureus* isolates from DFIs

Non-toxin virulence factor genes				Pooled estimates			Heterogeneity test	
	N	n_1_	n_2_	Proportion (%)	95% CI (%)		I^2^ (%)	
*bbp*	6	508	267	54.6	(20.3,86.8)		98.2	
*cna*	6	508	192	45.3	(21.8,69.9)		96.2	
*ebps*	6	508	234	70.7	(25.9,99.6)		98.9	
*clfa*	7	549	299	79.8	(28.8,100.0)		99.3	
*clfb*	6	508	305	86.2	(46.9,100.0)		98.8	
*fib*	6	508	246	63.1	(31.8,89.5)		97.7	
*fnba*	5	485	219	73.3	(21.5,100.0)		99.2	
*fnbb*	6	508	159	35.7	(14.0,60.8)		96.3	
*cap5*	4	609	174	35.0	(13.4,60.3)		96.7	
*cap8*	4	609	179	44.6	(10.6,81.8)		98.6	
*agr1*	9	687	281	38.2	(17.7,60.9)		96.9	
*agr2*	7	566	102	19.8	(13.0,27.5)		74.6	
*agr3*	7	566	69	11.7	(6.6,17.8)		71.2	
*agr4*	7	566	31	3.5	(0.1,9.9)		86.8	
*icaa*	3	333	113	100.0	(95.6,100.0)		47.8	
*icad*	3	333	113	100.0	(95.6,100.0)		47.8	
*eno*	3	163	136	91.7	(70.9,100.0)		80.7	
*chp*	3	158	90	61.0	(40.8,79.4)		78.1	
*scn*	3	158	143	71.5	(26.6,99.8)		96.1	

N: number of studies, n1: total number of isolates in all of the studies that report the respective virulence factor; n2: sum of the number of the isolated bacteria that report the respective virulence factor; CI: confidence interval

### Publication bias

Funnel plots of standard error with the prevalence of *luk-SF* (Fig. S2) and Begg’s test (p = 0.080) show no evidence of publication bias. We did not draw funnel plot for virulence factors reported in lower than 10 studies. Results of Begg’s test for virulence factors with more than 4 studies including *luk-ED* (p = 0.260), *sea* (p = 0.618), tst (p = 0.536), *hlb* (p = 0.308), *hlg* (p = 1.0), *etA* (p = 0.548), *seb* (p = 0.707), *seg* (p = 0.734), *she* (p = 0.707) and *sek* (p = 0.613) did not imply for publication bias. Among nontoxic virulence factors, Begg’s test results for *clfa* (p = 1.0), *clfb* (p = 0.707), fib (p = 0.260), *fnbA* (p = 0.462), *fnbB* (p = 1.0), agr1 (p = 0.917), agr2 (p = 0.230), agr3 (p = 0.230) and agr4 (p = 1.0) as well did not show significant evidence of publication bias.

## Discussion

Diabetic foot ulcer is one of the most serious complications of diabetes, which significantly affects patients’ quality of life. It can quickly spread into deeper tissue areas and cause critical conditions. Considering that bacteria are always present in the wound environment, making the diagnosis of infection only on the basis of microbial culture may lead to inappropriate prescription of antibiotics, which in turn leads to an increase in the prevalence of resistance to antibiotics, especially methicillin resistant *S. aureus* (MRSA) [10]. Identification of the most prevalent virulence factors of *S. aureus* isolates from DFIs may contribute more to the pathogenesis and help distinguish colonization from infection.

There are controversial issues about the role of PVL in skin soft tissue infections caused by *S. aureus*. In this study, although Luk-SF (PVL) was reported in most of our included articles, the prevalence in DFIs was not high and significant. This observation is consistent with the results of Stapper et al. [16]. Consistent with our findings, Víquez-Molina reported low proportion of several virulence factor genes, including *pvl*, *etA*, *etB*, and *tsst* in the profile of *S. aureus* recovered from DFIs [21]. Therefore, our study suggests that PVL toxin may not play a crucial role in the pathogenesis of DFIs, nor may it serve to differentiate colonization from infection. Interestingly, an Iranian study reported an unusual high prevalence of *pvl* (pvl, 56% and *luk-ED* 100%) in DFIs [22]. This observation suggests that the prevalence of virulence factors may be region-specific.

Several studies have established the role of *luk-ED* in the pathogenesis of *S. aureus* isolates from clinical samples [34–36]. Vasquez et al. identified a domain critical for targeting the *Staphylococcus aureu*s LukED receptor and erythrocyte lysis [37]. Djahmi et al. reported a high prevalence of *luk-ED* (96.5%) among *S. aureus* isolates from DFIs. They also found that several virulence factors, including *sek*, *seq*, *lukED*, *fnbB*, *cap8* and *agr* group 1 genes, were significantly associated with MRSA strains [13]. Another study reported 100% *luk-ED* positivity in *S. aureus* isolates from DFIs [22]. Interestingly, Dunyach-Remy et al. found statistical significance in the prevalence of *luk-ED* from DFU and nares isolates compared to DFU alone. This may imply that *luk-ED* made a significant contribution to DFI pathogenicity [20]. We also found a high pooled estimate of the proportion of *luk-ED* (72%).

Although there were a few studies reported the frequency of intercellular adhesions, we found the most pooled proportion for *icaA* and *icaD* (100%). This could therefore indicate that these factors may play a role in the formation of the biofilm and the development of the infection.

On the other hand, from a microbiological perspective, distinguishing colonization from infection is one of the key challenges for clinicians in the treatment of DFIs. Misdiagnosis of colonization as an infection can lead to inappropriate antibiotic prescribing, which in turn leads to an increase in the prevalence of antibiotic resistance, particularly methicillin-resistant *S. aureus* (MRSA) [10]. Sotto et al. (2008) found that the combination of five genes, including sea, sei, *lukED*, *hlgv*, and *cap8* was useful as predictive markers for distinguishing uninfected diabetic foot ulcers (grade 1) from infected ones (grade 2–4) [11]. This may mean that the genetic profiles of infecting and colonizing *S. aureus* strains isolated from uninfected and infected diabetic foot ulcers are different. Therefore, by comparing genetic profile of the infecting and colonizing isolates, some virulence factors may be found that have specific role in DFI pathogenesis. In the present study, we found a high pooled estimate of the proportion of *luk-ED* (72%) and *hlgv* (79%), but in the case of *sea*, *sei* and *cap8*, we did not obtain the same consistent results. This discrepancy may be due to the fact that in our study each virulence factor was considered individually and not in combination with other virulence factors. Additionally, we did not compare infected and uninfected ulcers.

### Limitations and strengths

One of the limitations of this study is that few articles reporting separate results for infected and uninfected ulcers. Therefore, we only considered the results for the infected ulcers. We were also unable to analyze the prevalence of virulence factors associated with MSSA and MRSA because most studies did not separately report the frequency of virulence factors for these isolates. Furthermore, we were unable to analyze the proportion of virulence factors associated with the type of infection (monomicrobial or polymicrobial) because most studies had focused on monomicrobial ulcers. The other limitation is that although numerous virulence factors were examined in all included articles, some of them were mentioned in only one or two articles and therefore were not included in the meta-analysis. The significant heterogeneity among studies could limit the interpretation of the pooled estimates. However, we attempted to address the results of each individual study to compensate for this heterogeneity. Finally, based on reports arising from PCR methods, it is difficult to say that prevalent genes have prevalent expression in a physiological situation and play a specific role in the pathogenicity.

The strengths of this systematic review and meta-analysis are worth noting. It provides a systematic and comprehensive search of all original published studies reporting the proportion of virulence factor genes of *S. aureus* isolates from DFIs. Furthermore, it is the first meta-analysis to examine the prevalence of virulence factors associated with the specific infection caused by *S. aureus*.

## Conclusion

The results of the present study showed that among the toxin virulence factors, hemolysins (*hld* (100.0%), *hlg* (88.0%), *hla* (80.0%), *hlgv* (79.0%)) and *luk-ED* (72, 0%) and among the non-toxin virulence factors, *icaA* and *icaD* (100.0%) stand out as having the highest proportion in *S. aureus* isolates from DFIs. These prevalent genes may have the potential to evaluate as virulence factors involved in DFI pathogenesis. Finding these probable virulence factor genes can help control diabetic foot infection more effectively via anti-virulence therapy or preparation of multi-epitope vaccines.

Moreover, the present study suggests that an effective approach to better distinguish colonization from infection could be to assess the intrinsic virulence potential of infecting strain of isolated bacteria. Therefore, these genes could also be assessed as candidate biomarkers, using an oligonucleotide microarray, to differentiate colonization from infection.

Future studies are recommended to examine the proportion of these prevalent virulence factor genes in the colonizing *S. aureus* isolates to demonstrate their specificity for DFI pathogenicity.

### Electronic supplementary material

Below is the link to the electronic supplementary material.

**Supplementary Material 1**: Quality assessment of studies using JBI’s critical appraisal tools designed for prevalence studies

**Supplementary Material 2**: Funnel plot of positive luk-SF proportions

## Data Availability

The datasets used and/or analysed during the current study available from the corresponding author on reasonable request.

## Abbreviations

DFI: diabetic foot infections
MRSA: methicillin resistant Staphylococcus aureus
PSM: phenol-soluble modulins
PVL: Panton-Valentine leucocidinMSCRAMM:Microbial surface components recognizing adhesive matrix molecules
SpA: staphylococcal protein A
Fnbp: fibronectin-binding proteins
Clf: clumping factor
PRISMA: Preferred Reporting Items for Systematic Reviews and Meta-Analyses
JBI: Joanna Briggs Institute
IDSA: The Infectious Diseases Society of America
