# Supplementary material for: Proportion of toxin and non-toxin virulence factors of Staphylococcus aureus isolates from diabetic foot infection: a systematic review and meta-analysis
Source: BMC Microbiol. 2024 Jan 3;24:1. doi: 10.1186/s12866-023-03142-y (PMC10763345; doi:10.1186/s12866-023-03142-y)
Supplement: Supplementary file 1 — Supplementary Material 1: Quality assessment of studies using JBI’s critical appraisal tools designed for prevalence studies [file 12866_2023_3142_MOESM1_ESM.docx]

**Supplementary file**

**Table S1** Quality assessment of studies using JBI’s critical appraisal tools designed for prevalence studies

| **No.** | **study** | **Year** | **Q1** | **Q2** | **Q3** | **Q4** | **Q5** | **Q6** | **Q7** | **Q8** | **Q9** | **Overall score** | **Include** |
| --- | --- | --- | --- | --- | --- | --- | --- | --- | --- | --- | --- | --- | --- |
| 1 | Sotto, A. | 2007 | Y | Y | Y | Y | NA | Y | Y | Y | NA | 7 | ✓ |
| 2 | Sotto, A. | 2008 | Y | Y | Y | Y | NA | Y | Y | Y | NA | 7 | ✓ |
| 3 | Sotto, A. | 2012 | Y | Y | Y | Y | NA | Y | Y | Y | NA | 7 | ✓ |
| 4 | Djahmi | 2013 | Y | Y | Y | Y | NA | Y | Y | Y | NA | 7 | ✓ |
| 5 | Post, V. | 2014 | Y | Y | N | Y | NA | N | Y | Y | NA | 5 | ✓ |
| 6 | Paul, S. K. | 2014 | Y | Y | N | Y | NA | N | Y | Y | NA | 5 | ✓ |
| 7 | Stappers, M. H. | 2015 | Y | Y | Y | Y | NA | UC | Y | Y | NA | 6 | ✓ |
| 8 | Shettigar, K. | 2016 | Y | UC | Y | Y | NA | Y | Y | Y | NA | 6 | ✓ |
| 9 | Mottola, C. (a) | 2016 | Y | UC | N | Y | NA | Y | Y | Y | NA | 5 | ✓ |
| 10 | Pobiega, M. | 2016 | Y | UC | Y | Y | NA | N | Y | Y | NA | 5 | ✓ |
| 11 | Dunyach-Remy, C. | 2017 | Y | Y | Y | Y | NA | Y | Y | Y | NA | 7 | ✓ |
| 12 | Víquez-Molina, G. | 2018 | Y | Y | Y | Y | NA | Y | Y | Y | NA | 7 | ✓ |
| 13 | Matias, C. | 2018 | Y | N | NC | Y | NA | UC | Y | Y | NA | 4 | 🗶 |
| 14 | Lin, S. Y. | 2018 | Y | UC | Y | Y | NA | Y | Y | Y | NA | 6 | ✓ |
| 15 | Kananizadeh, P. | 2019 | Y | NC | Y | Y | NA | Y | Y | Y | NA | 6 | ✓ |
| 16 | Silva, V. | 2019 | Y | N | N | Y | NA | Y | Y | Y | NA | 5 | ✓ |
| 17 | Stańkowska | 2019 | UC | UC | NC | Y | NA | N | Y | Y | NA | 3 | 🗶 |
| 18 | Anwar, K | 2020 | Y | UC | N | Y | NA | Y | Y | Y | NA | 5 | ✓ |
| 19 | Al-Bakri, A. G. | 2021 | Y | N | Y | Y | NA | Y | Y | Y | NA | 6 | ✓ |
| 20 | Soares, R. S. | 2020 | Y | N | N | Y | NA | N | UC | Y | NA | 3 | 🗶 |
| 21 | Pouget, C. | 2021 | Y | N | N | N | NA | N | Y | Y | NA | 3 | 🗶 |

**Abbreviations:** Y Yes, N No, U Unclear, NA Not Applicable, Q Question. Overall score is calculated by counting the number of Ys in each row

Q1 = Was the sample frame appropriate to address the target population? Q2 = Were study participants sampled in an appropriate way? Q3 = Was the sample size adequate? Q4 = Were the study subjects and the setting described in detail? Q5 = Was the data analysis conducted with sufficient coverage of the identified sample? Q6 = Were valid methods used for the identification of the condition? Q7 = Was the condition measured in a standard, reliable way for all participants? Q8 = Was there appropriate statistical analysis? Q9 = Was the response rate adequate, and if not, was the low response rate managed appropriate
