# Supplementary figures and images for: Proportion of toxin and non-toxin virulence factors of Staphylococcus aureus isolates from diabetic foot infection: a systematic review and meta-analysis
Source: BMC Microbiol. 2024 Jan 3;24:1. doi: 10.1186/s12866-023-03142-y (PMC10763345; doi:10.1186/s12866-023-03142-y)

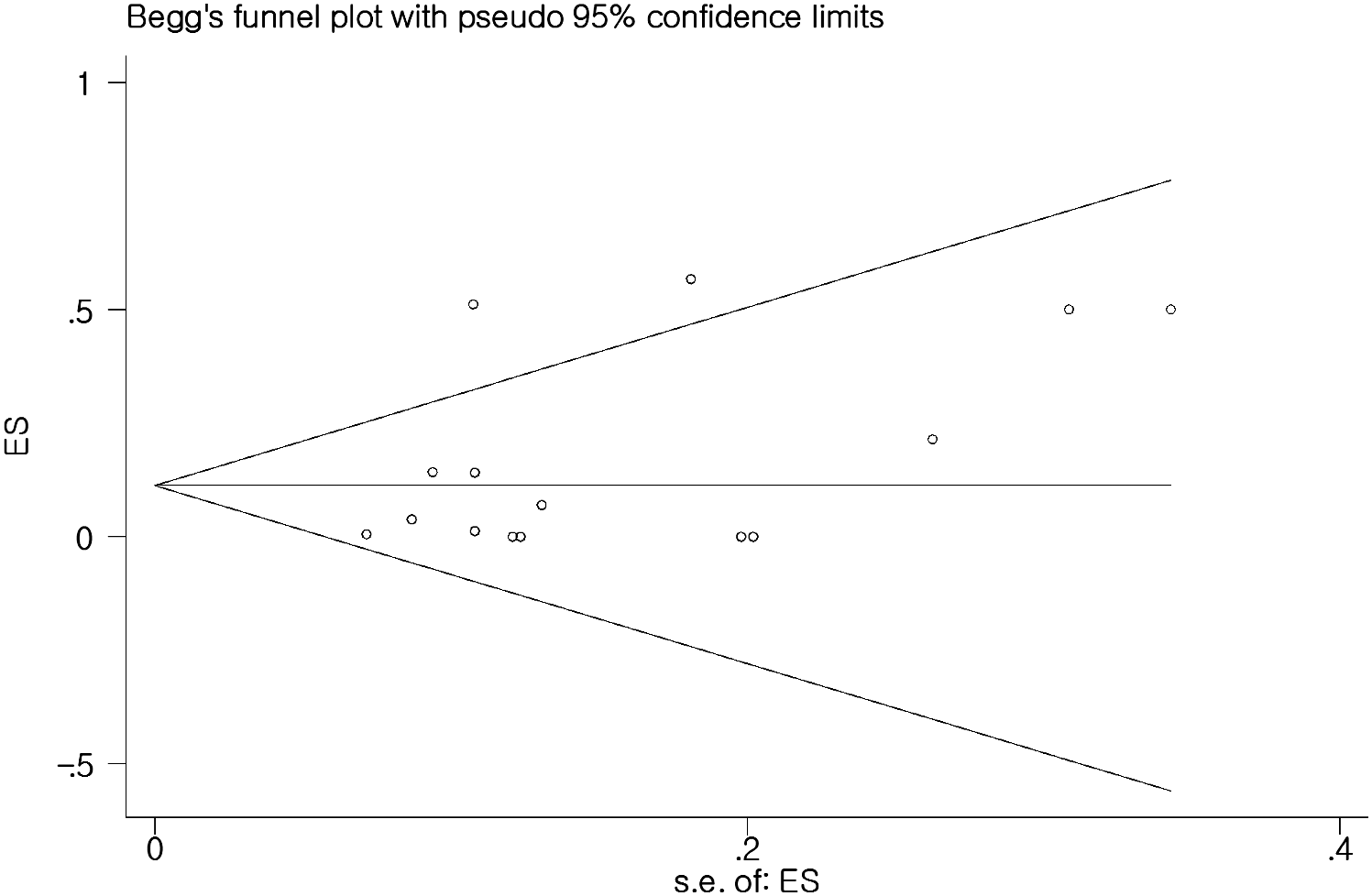

Supplement: Supplementary file 2 — Supplementary Material 2: Funnel plot of positive luk-SF proportions [file 12866_2023_3142_MOESM2_ESM.png]
